# Targeting SIRT2 Sensitizes Melanoma Cells to Cisplatin via an EGFR-Dependent Mechanism

**DOI:** 10.3390/ijms22095034

**Published:** 2021-05-10

**Authors:** Iwona Karwaciak, Anna Sałkowska, Kaja Karaś, Jarosław Dastych, Marcin Ratajewski

**Affiliations:** 1 Laboratory of Transcriptional Regulation, Institute of Medical Biology, Polish Academy of Sciences, 93-232 Lodz, Poland; isachrajda@cbm.pan.pl; 2 Laboratory of Epigenetics, Institute of Medical Biology, Polish Academy of Sciences, 93-232 Lodz, Poland; salkowska@cbm.pan.pl (A.S.); kkaras@cbm.pan.pl (K.K.); 3 Laboratory of Cellular Immunology, Institute of Medical Biology, Polish Academy of Sciences, 93-232 Lodz, Poland; jdastych@cbm.pan.pl

**Keywords:** melanoma, SIRT2, cisplatin, EGFR, resistance

## Abstract

Melanoma cells are resistant to most anticancer chemotherapeutics. Despite poor response rates and short-term efficacy, chemotherapy remains the main approach to treating this cancer. The underlying mechanisms of the intrinsic chemoresistance of melanoma remain unclear, but elucidating these mechanisms is important to improve the efficacy of chemotherapy regimens. Increasing evidence suggests that sirtuin 2 (SIRT2) plays a key role in the response of melanoma cells to chemotherapeutics; thus, in the present study, we evaluated the impact of shRNA-mediated and pharmacological inhibition of SIRT2 on the sensitivity of melanoma cells to cisplatin, which is used in several regimens to treat melanoma patients. We found that cells with SIRT2 inhibition revealed increased sensitivity to cisplatin and exhibited increased accumulation of γ-H2AX and reduced EGFR-AKT-RAF-ERK1/2 (epidermal growth factor receptor-protein B kinase–RAF kinase-extracellular signal-regulated kinase 1/2) pathway signaling compared to control cells. Thus, our results show that sirtuin 2 inhibition increased the in vitro efficacy of cisplatin against melanoma cells.

## 1. Introduction

While melanoma is a relatively rare skin neoplasm, it is also the most dangerous skin neoplasm, accounting for the majority of deaths caused by this tumor type [1]. The incidence of melanoma has steadily increased and is potentially associated with UV exposure, leading to oxidative stress, DNA damage, mutations, and as consequently, melanomagenesis [2].

A major challenge in the treatment of malignant melanoma is the high resistance of melanoma cells to chemotherapeutics. The high drug resistance of melanomas may be related to metabolic processes involved in melanin production by the melanocytes, from which melanoma originates. These processes generate the endogenous melanogenic cytotoxicity (EMC) phenomenon, which leads to the adaptation of melanocytes to high concentrations of cytotoxic compounds by the development of detoxification mechanisms [3,4]. However, it should be noted that numerous other unknown mechanisms are involved in multidrug resistance of melanoma cells (intrinsic and acquired) that subsequently lead to poor response rates and low efficacy of chemotherapeutic treatment. This resistance is considered to be the main cause of the failure of standard or targeted therapies against melanoma and other cancers [5]. Therefore, it is important to elucidate the processes responsible for resistance to a specific drug, as this may provide potential therapeutic targets and improve the efficacy of known therapies.

Several reports suggest that sirtuins are involved in the resistance of melanoma to several anticancer drugs [6,7,8,9] and are promising targets in potential treatment strategies against melanoma [10,11,12]. To date, the most commonly used drugs to treat melanoma have been alkylating agents, dacarbazine and temozolomide [13,14,15]; however, the partial and complete responses are not satisfying. Thus, to improve their efficacy, other forms of cancer therapy, such as IL-2 or INFα, are typically added [15]. Cisplatin is considered to be a DNA (genomic and mitochondrial)-binding drug, creating adducts with DNA, damaging it, and leading to necrosis or apoptosis. However, in addition to cisplatin’s action on DNA, this drug also modifies proteins and gene expression and influences signaling involved in cell survival and proliferation [16]. Cisplatin, which is used to treat several types of cancers (e.g., small cell lung cancer, ovarian cancer, bladder cancer, testicular cancer, cervical cancer, and gastric cancer [17]), is part of several regimens of combined therapy against melanoma, e.g., cisplatin, vinblastine, and dacarbazine (CVD regimen), as well as cisplatin, dacarbazine, carmustine, and tamoxifen (Dartmouth regimen) [15,18,19,20]. However, cisplatin usage is limited by its toxic side effects and the acquisition of resistance that appears during treatment [17].

Here, using several methods, we demonstrate that melanoma cells with knockdown of sirtuin 2 (SIRT2) are more susceptible to cisplatin, suggesting that this sirtuin might be involved in the mechanisms of melanoma resistance to this drug [21]. The mechanistic effect of sirtuin 2 inhibition on melanoma cell resistance may involve the epidermal growth factor receptor (EGFR), which we previously found to be directly regulated by SIRT2 in melanoma cells [9], and a decrease in AKT-RAF-ERK1/2 (protein B kinase–RAF kinase-extracellular signal-regulated kinase 1/2) signaling phosphorylation. The consequence of our study is that targeting SIRT2 (with specific inhibitors) may potentially increase the efficacy of cisplatin in regimens currently used for melanoma management.

## 2. Results

### 2.1. SIRT2 Downregulation Increases the Susceptibility of Melanoma Cells to Cisplatin

**Figure 1:**
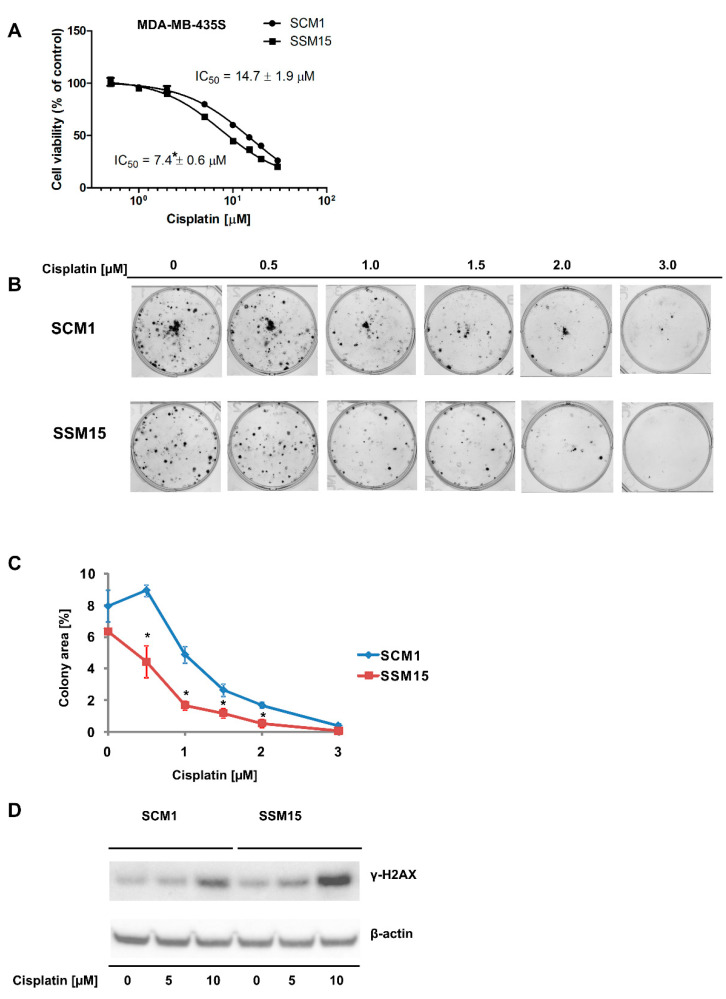
shRNA-mediated downregulation of SIRT2 sensitizes melanoma cells to cisplatin. (**A**) The effects of cisplatin treatment (96 h) on the viability of melanoma SCM1 and SSM15 clones of the MDA-MB-435S cell line were evaluated using the neutral red assay. The results are shown as the mean ± standard deviation (*n* = 3, independent experiments). (**B**) Results of the colony formation assay performed on melanoma SCM1 and SSM15 clones of the MDA-MB-435S cell line exposed to different cisplatin concentrations. Images from a single representative experiment are shown. (**C**) A colony area values from the colony forming assay shown as the mean ± standard deviation (*n* = 3, independent experiments). * Indicates a statistically significant difference at *p* < 0.05. (**D**) Effect of cisplatin on the accumulation of γ-H2AX in melanoma SCM1 and SSM15 clones of the MDA-MB-435S cell line. Cells were treated with cisplatin for 48 h. Then, cells were harvested and protein lysates were prepared and analyzed by Western blotting.

Our previous analysis of the transcriptomes of cells that differ in SIRT2 levels (Figure S1): MDA-MB-435S SCM1 and MDA-MB-435S SSM15 revealed the altered expression of numerous genes involved in cellular signaling and DNA repair (e.g., *EGFR*, *EPHA2*, *ZNF365*, and *NT5E*) [9]. Thus, we hypothesized that cells with low SIRT2 expression would be more sensitive to cisplatin. The results of the neutral red viability assay showed increased cisplatin cytotoxicity in melanoma cells with downregulated SIRT2 expression (Figure 1A). A colony formation assay performed to confirm our initial results also showed that MDA-MB-435S SSM15 cells were more sensitive to cisplatin (Figure 1B,C). Furthermore, cisplatin induced more γ-H2AX, which is considered an indicator of DNA double-strand breaks [22], in MDA-MB-435S SSM15 cells compared with MDA-MB-435S SCM1 cells expressing normal SIRT2 levels (Figure 1D). This might be mediated by the downregulation of DNA repair involved genes: *ZNF365* and *NT5E* [23,24] is observed in SIRT2 silenced cell line (Figure S2), and thus SIRT2 may have a protective functions against cisplatin-induced cytotoxicity by promoting nucleotide excision repair, as was previously reported by Zhang et al. [25].

**Figure 2:**
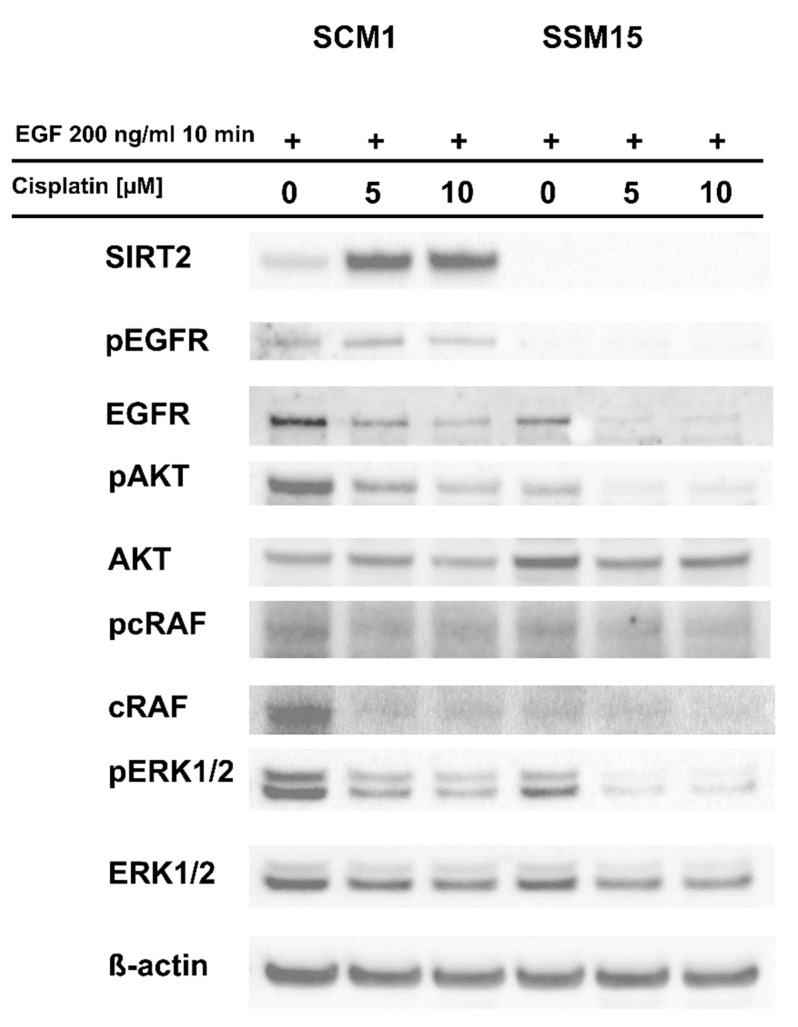
SIRT2 downregulation impairs the response of melanoma cells to EGF and cisplatin. The generated melanoma clones (SCM1 and SSM15 clones) were treated with selected concentrations of cisplatin for 48 h and then treated with EGF (EGFR activator) for 10 min, and protein lysates were prepared and analyzed by Western blotting.

A previous study showed that SIRT2 regulates the expression of several tyrosine kinase receptors, including *EGFR* (epidermal growth factor receptor), and affects downstream signaling of SRC-ERK (proto-oncogene tyrosine-protein kinase Src-extracellular signal-regulated kinase) [9], which plays a prominent role in the proliferation and resistance of melanoma to certain drugs [26,27]. This finding prompted us to investigate the effects of cisplatin on EGF-dependent phosphorylation of EGFR and downstream elements of this signaling pathway. As shown in Figure 2, EGFR phosphorylation was lower in cells exposed to EGF, in which SIRT2 was downregulated, and the effect was further decreased by cisplatin. EGFR protein expression was also downregulated by cisplatin (Figure 2 and Figure S3). As the effect of the drug on pSRC was rather minimal (data not shown), we decided to assess other signaling pathways downstream of EGFR, which are crucial for melanoma cell survival, including pAKT, pcRAF and pERK1/2 [28]. As shown in Figure 2, AKT, cRAF and ERK1/2 phosphorylation was substantially diminished after treatment with cisplatin, and this decrease was more evident in cells with *SIRT2* knockdown. Interestingly, the expression of SIRT2 protein was substantially increased in the SCM1 clone by cisplatin, indicating that this protein can play a prominent role in the drug resistance of melanoma cells to this drug (Figure 2 and Figure S3).

### 2.2. Pharmacological Inhibition of Sirtuin 2 Increases the Susceptibility of Melanoma Cells to Cisplatin

**Figure 3:**
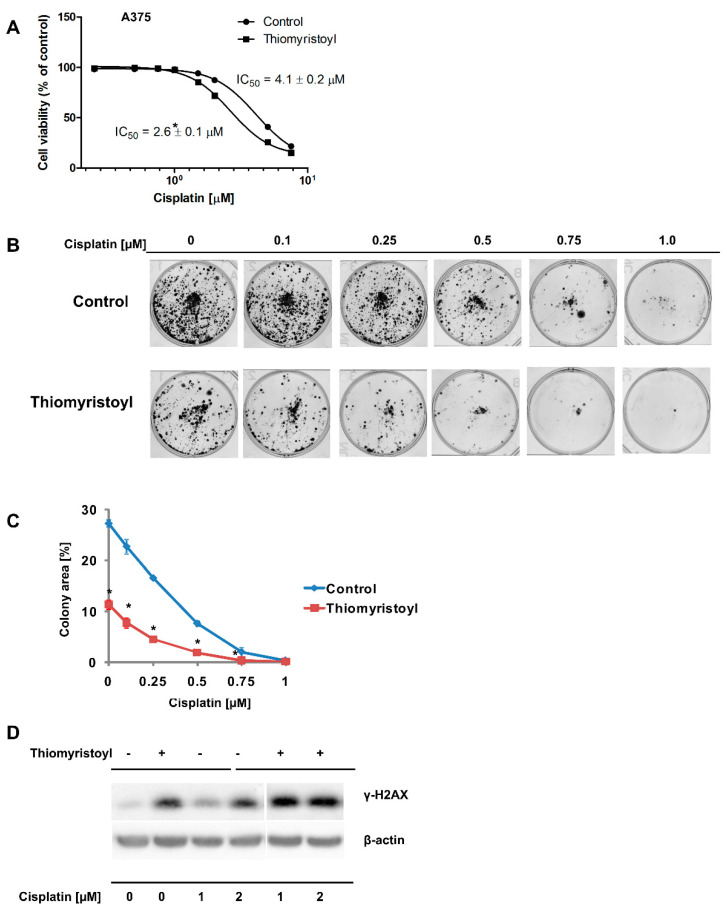
Pharmacological inhibition of SIRT2 sensitizes melanoma A375 cells to cisplatin. (**A**) The effects of cisplatin treatment (96 h) on the viability of control cells and those pretreated with thiomyristoyl cells were determined using the neutral red assay, mean ± standard deviation (*n* = 3, independent experiments). (**B**) Results of the colony formation assay performed on control A375 cells and those pretreated with thiomyristoyl for 72 h prior to exposure to different cisplatin concentrations. Images from a single representative experiment are shown. (**C**) A colony area values from the colony forming assay is shown as the mean ± standard deviation (*n* = 3, independent experiments). * Indicates a statistically significant difference at *p* < 0.05. (**D**) Effect of cisplatin on the accumulation of γ-H2AX in control A375 cells and those pretreated with thiomyristoyl for 72 h. Cells were treated with cisplatin for 48 h. Then, cells were harvested and protein lysates were prepared and analyzed by Western blotting.

**Figure 4:**
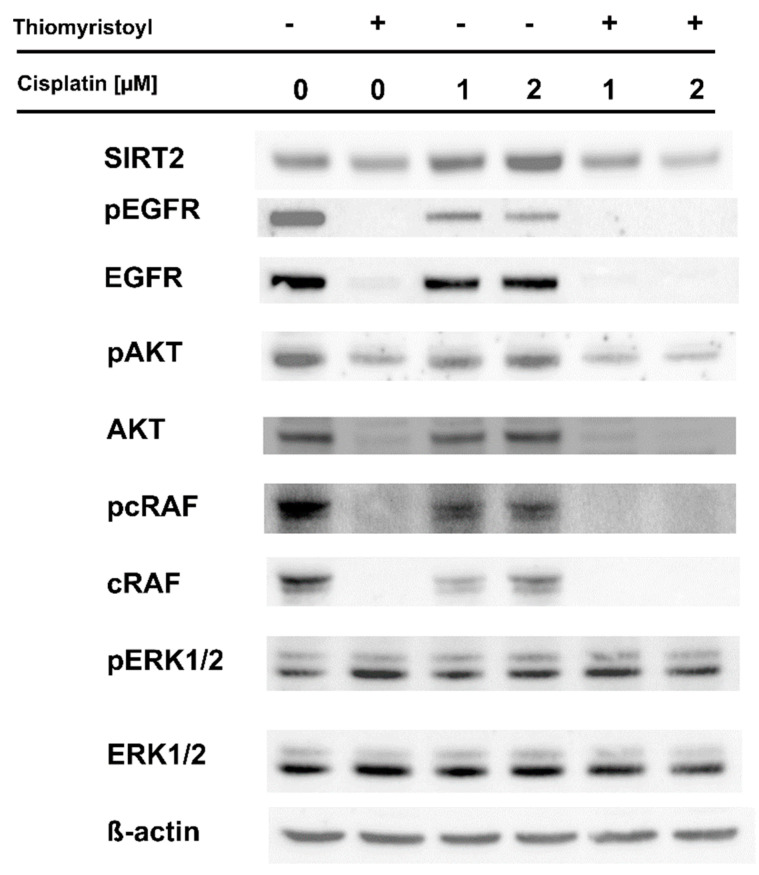
SIRT2 pharmacological inhibition impairs the response of melanoma cells to cisplatin. Control A375 cells and those pretreated with thiomyristoyl were treated with selected concentrations of cisplatin for 48 h. Then, protein lysates were prepared and analyzed by Western blotting.

Next, we analyzed the effects of pharmacological inhibition of sirtuin 2 on cisplatin activity against a metastatic melanoma cell line with intact sirtuin 2 expression. In this series of experiments, we employed the A375 cell line and thiomyristoyl [29], an inhibitor that affects both the deacetylation and demyristolylation activities of sirtuin 2 [30]. We observed that A375 cells pretreated with thiomyristoyl were more sensitive to cisplatin treatment in the neutral red viability assay (Figure 3A) and a colony-forming assay (Figure 3B,C). Cells treated with thiomyristoyl exhibited higher levels of γ-H2AX, and this effect of sirtuin 2 inhibition was enhanced by cisplatin (Figure 3D). Analysis of EGFR phosphorylation indicated that similar to MDA-MB-435S cells, A375 cells had less EGFR after cisplatin treatment than control cells and had increased SIRT2 levels (Figure 4 and Figure S4). As expected, inhibition of sirtuin 2 activities resulted in a decrease in EGFR levels (Figure 4 and Figure S4), confirming our previous observation that this sirtuin regulates *EGFR* gene expression in melanoma cells [9]. Interestingly, thiomyristoyl inhibited AKT and cRAF expression, which was associated with a decrease in the phosphorylation status of these signaling proteins. In contrast to MDA-MB-435S cells, we did not observe the effects of thiomyristoyl and cisplatin on pERK1/2 in A375 cells (Figure 4 and Figure S4). This discrepancy might be caused by the differences between the signal transduction in different cell lines (MDA-MB-435S and A375) or off-target effects of the SIRT2 inhibitor.

## 3. Discussion

In recent years, the prognosis for patients with advanced melanoma has improved; immunotherapy and targeted therapies (e.g., with V600E BRAF mutant-specific inhibitors [31]) have resulted in significant increases in median overall survival [32,33,34]. However, the efficacy of single-agent chemotherapy and combined chemotherapy remains unsatisfactory due to a high level of drug resistance [34]. Therefore, it is crucial to identify and understand the mechanisms underlying drug resistance to develop new combinations of existing drugs with better therapeutic effects and to identify novel molecular therapeutic targets. A growing number of studies indicate that class III histone deacetylases are important players in the development, progression, and drug resistance of skin cancers, including melanoma [11,12,35]. Our previous studies showed that pharmacological inhibition of SIRT2 and stable shRNA-mediated knockdown sensitized melanoma cells to doxorubicin and dasatinib, respectively [6,9], and analysis of transcriptomes revealed that sirtuin 2 is an important regulator of genes involved in melanoma progression and drug resistance (e.g., integrins, tyrosine kinase receptors, MAP kinases, etc.) [9]. Here, compared to control cells, cells with SIRT2 downregulation were more susceptible to cisplatin treatment (Figure 1A–C), accumulated greater γ-H2AX amounts (Figure 1D) which was associated with the decreased levels of DNA repair genes (Figure S2), and exhibited different phosphorylation profiles of EGFR and downstream signaling elements, i.e., AKT-RAF-ERK1/2 (Figure 2 and Figure S3). Furthermore, we confirmed these results in another metastatic cell line (A375) in which sirtuin 2 activity was inhibited by thiomyristoyl (Figure 3, Figure 4 and Figure S4). We hypothesize that the observed enhancement of the effects of cisplatin in cells with decreased sirtuin 2 activity is mediated by increased degradation of EGFR protein by this compound, as shown previously [36,37]. Given that there was less EGFR in cells with *SIRT2* knockdown, the effect of the drug was more prominent compared to control cells. However, we could not exclude the fact that additional known mechanisms of cisplatin resistance, such as inactivation of drugs by modification, increased the expression of antiapoptotic proteins, overexpression of DNA repair proteins and increased DNA repair capacity [17], are affected by sirtuin 2 inhibition.

SIRT2 is NAD(+)-dependent lysine deacetylase that localizes in both the cytoplasm and nucleus and plays a prominent role in the regulation of nervous system development, metabolism, and the cell cycle [38,39,40,41]. In cancer, depending on the type and stage of the disease, this sirtuin may act as a tumor promoter and/or suppressor [41,42]. In an elegant work, Wang et al. demonstrated that high SIRT2 levels are associated with the sensitivity of ovarian cancer cells to cisplatin, thus confirming the complexity of SIRT2 functions depending on cancer type [43].

## 4. Materials and Methods

### 4.1. Cell Lines, Cell Culture and Reagents

MDA-MB-435S SCM1 (control) and MDA-MB-435S SSM15 (downregulated SIRT2, stage metastatic) cells were generated by stable transfection of the maternal MDA-MB-435S cell line (ATCC, Manassas, VA, USA) with scrambled negative control shRNA (TR30012) or SIRT2 (T301692D) (Origene Tech., Rockville, MD, USA) as previously described [9]. The human melanoma cell line A375 (stage metastatic) was obtained from ATCC (Manassas, USA) and was maintained in Dulbecco’s modified Eagle’s medium supplemented with 10% fetal bovine serum (PAN-Biotech GmbH, Aidenbach, Germany). All cells were cultured at 37 °C in a humidified atmosphere containing 5% CO2. Cisplatin was purchased from Sigma Aldrich (St. Louis, MO, USA). Human EGF was purchased from Sigma Aldrich (St. Louis, MO, USA). Thiomyristoyl was purchased from Cayman Chemical (Ann Arbor, MI, USA).

### 4.2. Cell Viability Measurements

Cell viability was determined using the neutral red assay [44]. Cells were seeded on 96-well transparent plates at a density of 4000 cells per well. Twenty-four hours later, cells were treated with increasing concentrations of cisplatin for 96 h, neutral red was added for 2 h, and plates were processed as described in our previous study [45].

### 4.3. Colony Formation Assay

Cells were seeded in a 6-well plate at a density of 750 cells per well. Twenty-four hours later, the cells were treated with increasing concentrations of cisplatin for 10–14 days. Then, cellular colonies were fixed with 100% methanol and stained with 0.5% crystal violet solution as described elsewhere [46]. A G-Box documentation system (Synoptics, Cambridge, UK) was used to scan the plates, and the obtained images were analyzed using ImageJ as previously described [47].

### 4.4. RNA Extraction, Reverse Transcription and Quantitative PCR

TRI reagent (Sigma Aldrich, St. Louis, MO, USA) was used for cell lysis. Total RNA was extracted using Chomczynski method [48]. 5 μg of RNA was reverse transcribed using the Maxima First Strand cDNA Synthesis Kit for RT-quantitative PCR (Thermo Fisher Scientific, Waltham, MA, USA). Quantitative PCR was performed using SYBR Green I Master Mix (Roche, Basel, Switzerland) and reactions were run on a LightCycler 480 from Roche (Basel, Switzerland). The following conditions were used: 95 °C for 5 min, followed by 40 cycles of 95 °C for 10 s, 60 °C for 10 s, and 72 °C for 20 s. The following primers were used: *SIRT2*, 5′-GAAGGACAAGGGGCTACTCC-3′ (forward) and 5′-GATATCAGGCTTCACCAGGC-3′ (reverse) described previously [9], *ZNF365*, 5′-GACGGAATCTGAGGAGGAGC-3′ (forward) and 5′-ATCACGGACAAAGCCAGAGG-3′ (reverse); *NT5E*, 5′-TCACTTCTGATGATGGGCGG-3′ (forward) and 5′-AATCAGGTTGCCCATGTTGC-3′ (reverse) The mRNA levels were normalized by the *RPL13A* housekeeping gene, 5′-CCTGGAGGAGAAGAGGAAAGAGA-3′ (forward), and 5′-TTGAGGACCTCTGTGTATTTGTCAA-3′ (reverse), as described by Vandensompele et al. [49].

### 4.5. DNA Damage

To evaluate the DNA damage mediated by cisplatin, MDA-MB-435S SCM1 and MDA-MB-435S SSM15 cells were seeded on 6-well plates. On the next day, cells were treated with 5 and 10 μM cisplatin for 48 h. A375 cells (control and pretreated with thiomyristoyl, 50 μM, 72 h) were seeded on 6-well plates. On the following day, cells were treated with 1 and 2 μM cisplatin for 48 h. Afterwards, the cells were harvested and lysed, and Western blotting was performed to detect the amount of histone γ-H2AX using γ-H2AX antibody (ab11174, Abcam, Cambridge, UK).

### 4.6. Western Blotting

Proteins from cells were extracted using ice-cold RIPA buffer (50 mM Tris-HCl pH 8.0, 150 mM NaCl, 0.1% Triton X-100, 0.1% SDS, 0.5% sodium deoxycholate) containing Halt Protease Inhibitor Cocktail (Thermo Fisher Scientific, Waltham, MA, USA). The Pierce BCA Protein Assay kit (Thermo Fisher Scientific) was used to determine the protein concentration in the samples. Proteins were separated on a 12% Bis–Tris NuPage precast gel (Thermo Fisher Scientific). The iBlot dry blotting system (Thermo Fisher Scientific) was used to transfer proteins to a Hybond-C membrane (GE Healthcare Life Sciences, Marlborough, MA, USA). The membranes were then blocked with 5% milk and incubated overnight with primary antibodies at 4 °C. Detection was subsequently performed with HRP-conjugated secondary antibody (ab6721, Abcam, Cambridge, UK). We used the following primary antibodies: anti-SIRT2 (EPR1667) (Abcam), anti-beta actin (Abcam), EGF receptor (Cell Signaling, Danvers, MA, USA), phospho-EGF receptor (Tyr1068) (D7A5) (Cell Signaling), phospho-c-Raf (Ser338) (56A6) (Cell Signaling), c-Raf antibody (Cell Signaling); AKT1 antibody (GeneTex, Irvine, CA, USA); phospho-AKT (Ser473) (GeneTex); ERK 1/2 antibody (C-9) (Santa Cruz, Dallas, TX, USA), and phospho-ERK (E-4) (Santa Cruz). SuperSignal West Pico Chemiluminescent Substrate was used (Thermo Fisher Scientific) for the detection of specific bands. Membranes were then scanned using the G-Box chemiluminescence imaging station (Syngene, Cambridge, UK).

### 4.7. Statistics

Tests for statistical significance were performed using ANOVA followed by Tukey’s post hoc test. When *p* < 0.05, the observed difference was considered statistically significant.

## 5. Conclusions

In conclusion, our study [9], and others [50,51], suggest that SIRT2 is a tumor promoter in melanoma cells and this sirtuin is a promising target in antimelanoma therapy; these levels may influence the overall survival of melanoma patients [51]. The present study shows that inhibition of SIRT2 (shRNA-mediated knockdown and pharmacological by thiomyristoyl) resulted in sensitization of melanoma cells to the widely used anticancer drug cisplatin, most likely by targeting EGFR stability and downstream signaling. We thus propose that the efficacy of currently used regimens in antimelanoma therapy involving cisplatin could be improved by SIRT2 inhibition.

## Data Availability

All data for this study are included in the manuscript and the supplementary files.

## Supplementary Materials

The following are available online at https://www.mdpi.com/article/10.3390/ijms22095034/s1, Figure S1. Analysis of SIRT2 expression in MDA-MB-435S cellular clones: SCM1 and SSM15; Figure S2. Analysis of expression of the genes involved in DNA repair in MDA-MB-435S cellular clones: SCM1 and SSM15; Figure S3. Results of densitometric analysis of the blots presented in Figure 2; Figure S4. Results of densitometric analysis of the blots presented in Figure 4; Original scans of blots presented in the Figure 1, Figure 2, Figure 3 and Figure 4.
