# Supplementary material for: Targeting SIRT2 Sensitizes Melanoma Cells to Cisplatin via an EGFR-Dependent Mechanism
Source: Int J Mol Sci. 2021 May 10;22(9):5034. doi: 10.3390/ijms22095034 (PMC8126047; doi:10.3390/ijms22095034)
Supplement: Supplementary file 1 [file ijms-22-05034-s001.zip › ijms-1182118-supplementary.pdf]

Supplementary materials

Targeting SIRT2 sensitizes melanoma cells to cisplatin via an EGFR-dependent mechanism.

Iwona Karwaciak, Anna Sałkowska, Kaja Karaś, Jarosław Dastych, and Marcin Ratajewski

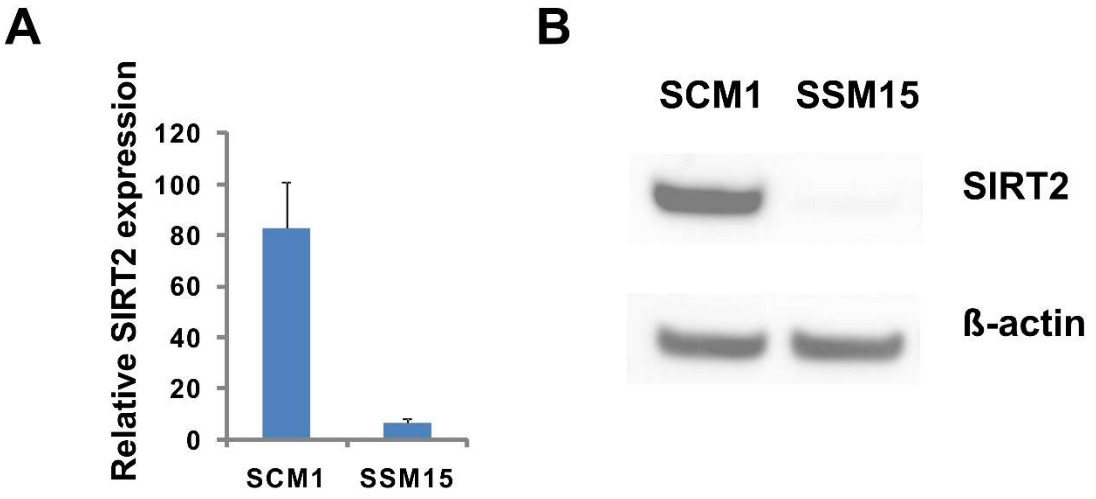

Figure S1. Analysis of SIRT2 expression in MDA-MB-435S cellular clones: SCM1 and SSM15. (A) Expression of mRNA of SIRT2 gene evidenced by real time RT-PCR, mean ± SD, n = 3. (B) SIRT2 expression evidenced by Western blotting.

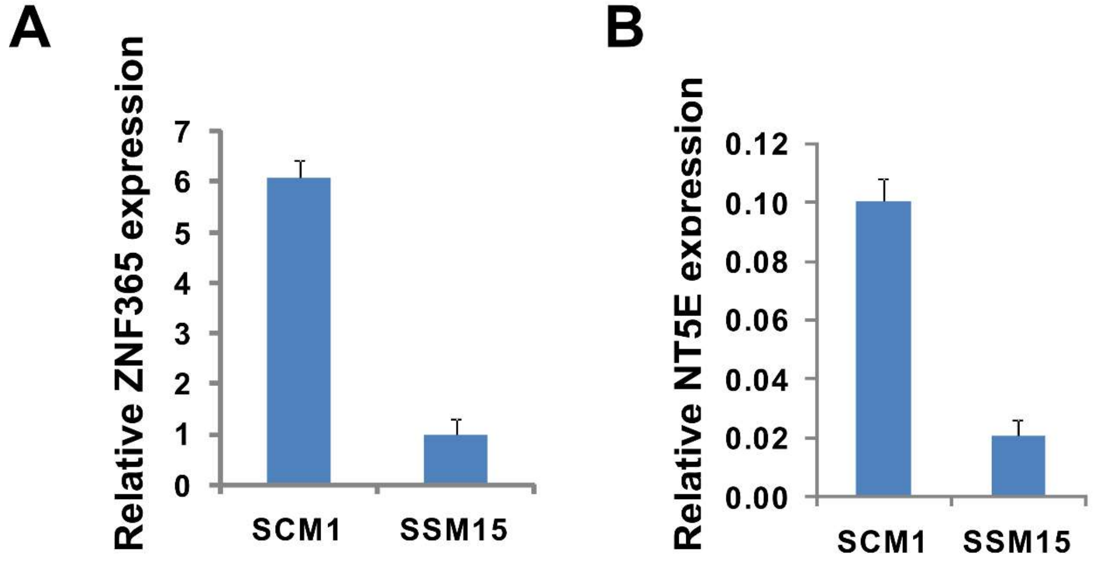

Figure S2. Analysis of expression of the genes involved in DNA repair in MDA-MB-435S cellular clones: SCM1 and SSM15. (A) *ZNF365* mRNA expression evidenced by real time RT-PCR, mean ± SD, n = 3. (B) *NT5E* mRNA expression evidenced by real time RT-PCR, mean ± SD, n = 3.

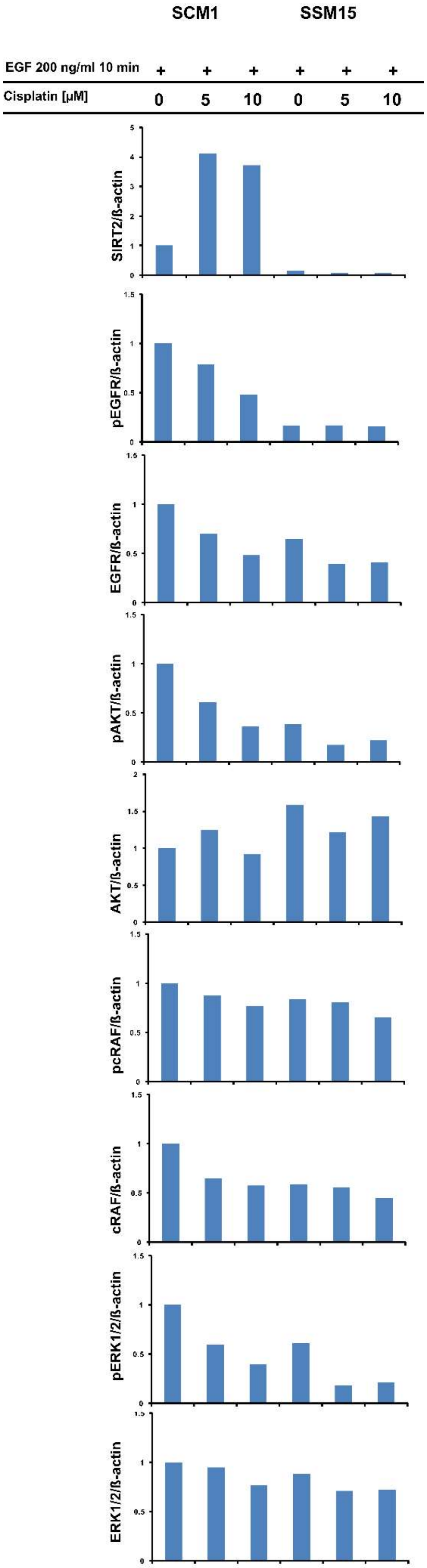

Figure S3. Results of densitometric analysis of the blots presented in Fig. 2. Blots were analyzed using SysTools from Syngene. .

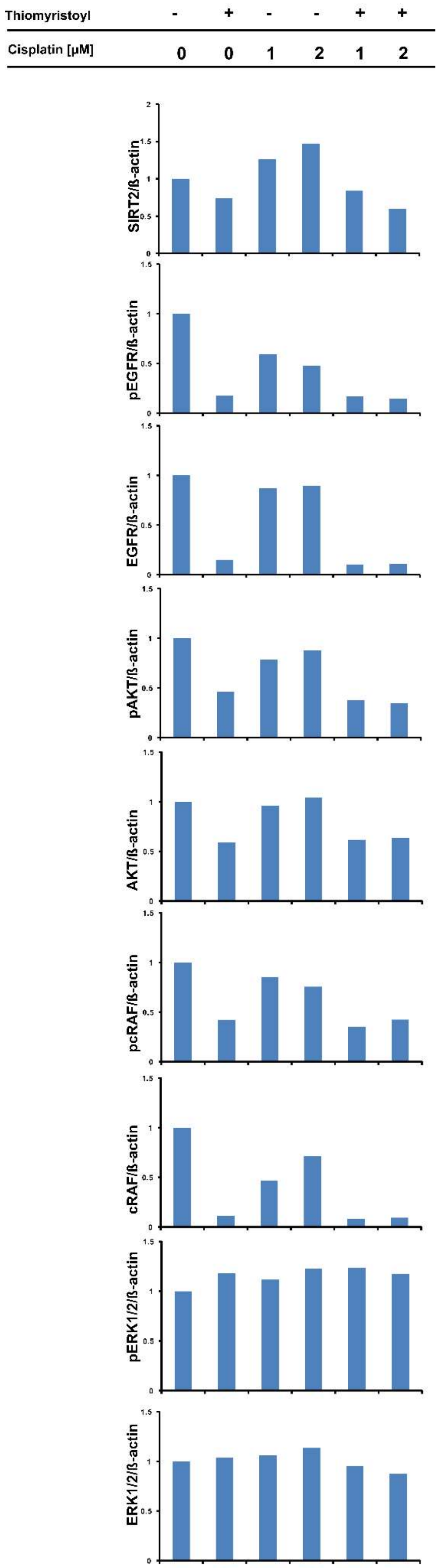

Figure S4. Results of densitometric analysis of the blots presented in Fig. 4. Blots were analyzed using SysTools from Syngene.
